# Public health effects of gambling – debate on a conceptual model

**DOI:** 10.1186/s12889-019-7391-z

**Published:** 2019-08-09

**Authors:** Tiina Latvala, Tomi Lintonen, Anne Konu

**Affiliations:** 1 Finnish Foundation for Alcohol Studies, P.O. Box 30, FI-00271 Helsinki, Finland; 2 Faculty of Social Sciences, Health Sciences, Tampere University, FI-33014 Tampere, Finland

**Keywords:** Public health, Gambling, Conceptual model

## Abstract

**Background:**

Gambling for money is a popular leisure time activity in most countries, which has major social and economic impacts not only affecting the gambler, but his/her significant others, and the society. Gambling impact studies can help researchers and policymakers compare the health and social costs and benefits of different gambling policies and can be used when considering which gambling policies will reduce or increase costs or benefits the most. In a public health approach, the impacts of gambling, negative and positive, are assessed across the entire severity spectrum of the activity. Although some studies have created basic principles for conducting impact studies, a theoretical model is currently lacking. The aim of this debate is to review complementing and contrasting views on the effects of gambling to create a conceptual model, where a public health perspective is applied.

**Main text:**

The effects of gambling can be structuralized using a conceptual model, where impacts are divided into negative and positive; costs and benefits. Costs and benefits are categorized into three classes: financial, labor and health, and well-being. These classes manifest in personal, interpersonal, and societal levels. Individual impacts cause effects on a personal level to gamblers themselves. External impacts influence the interpersonal and society/community levels and concern other people. The temporal level refers to the development, severity and scope of the gambling impact. These include general impacts, impacts of problem gambling and long-term impacts of gambling.

**Conclusions:**

The conceptual model offers a base on which to start building common methodology for assessing the impact of gambling on the society. While measuring monetary impacts is not always straightforward, the main issue is how to measure the social impacts, which are typically ignored in calculations, as are personal and interpersonal impacts. The reviewed empirical work largely concentrated on the costs of gambling, especially costs on the community level. The Model can be used to identify areas where research is scarce. Filling the gaps in knowledge is essential in forming a balanced evidence base on the impacts of gambling. Ideally, this evidence could be the starting point in formulating public policies on gambling.

## Background

Gambling can be defined as betting money on an outcome of uncertain results to win money. All forms of gambling, even those typically considered to be more skill-based, like poker and sports betting, contain an element of luck [1]. Another common characteristic of gambling is that it is a zero-sum game: when one player wins, the other must lose [2]. Gambling is a popular leisure time activity in most countries, and the vast majority of adults have engaged in some gambling activity at least once in their life, and between 40 and 80% have participated in some form of gambling in the last 12 months [3]. For most individuals, gambling is a form of entertainment [4, 5]. For some consumers, the motivation for gambling is influenced by social interactions because gambling venues offer social settings to meet people [6, 7], whereas others are mainly motivated by the dream of winning money [8]. By contrast, some use gambling to escape their problems, and this is especially common among problem gamblers [9].

Gambling is typically viewed as a continuum, with most people gambling only occasionally or not at all and some gambling more frequently. Along this continuum, people can experience negative financial and social consequences, although harms tend to be more common among frequent gamblers [10]. Based on harms experienced because of gambling, gamblers are usually divided to recreational, at-risk, and problem and pathological gamblers [11, 12]. Problem and pathological gamblers are usually called problematic gamblers. Pathological gambling is a disorder included in both diagnostic manuals: International Classification of Disorders [13] and Diagnostic and Statistical Manual [14]. Prevalence of problem and pathological gambling varies between countries, but it is estimated that among adult population 1 to 4% are problem gamblers [15], whereas prevalence estimates of pathological gambling range from 0.1 to 0.8% [16]. There are, however, much more people suffering from gambling-related harms.

Harms caused by gambling can co-occur with other difficult situations in life, usually intensifying along with crises and continuing even after the problematic behavior comes to an end [17, 18]. Gambling-related harm can affect multiple domains of life [17], including financial [19, 20] and health problems [21, 22], psychological and emotional distress [23, 24], and impaired social and cultural relationships [25–27]. They have an influence on multiple levels: gambling-related harms restrict the gambler and their family, friends, workplace, community, and society [17, 18, 28, 29]. Because of these significant influences on society and the population’s overall health, gambling is a critical public health issue [30, 31].

The impacts of gambling on societies is positive and negative and depends on a number of factors, including what type of gambling environments and games are available, how long gambling has been possible, whether gambling revenues are derived locally or outside the jurisdiction, and the effectiveness of gambling policy [32–35]. Overall, there are several main purposes for conducting impact studies on gambling. First, to demonstrate that gambling has major social and economic impacts. Impact studies can also help researchers and policymakers compare the impact of different health and social problems and benefits; for example, gambling impacts can be weighed against alcohol impacts. Additionally, impact studies can be used when considering which gambling policies will reduce or increase costs or benefits the most [36].

Different approaches have been used to study the impacts of gambling. Research into the socioeconomic impacts of gambling can be conducted from a cost of illness perspective, commonly used in alcohol and drug research; however, this approach neglects the benefit side [37]. Economic cost–benefit analysis (CBA) measures changes in well-being in common units (dollars) [38] and attempts to discover whether increased gambling opportunities are positive for society [39]. In this approach, monetary value is also assigned to intangible harms (harms not necessarily monetary in nature, e.g., the pain and suffering of problem gambler), and harms are known to affect others in addition to the gamblers themselves. This approach, however, has been criticized because an arbitrary monetary value is applied to these intangible harms [37, 40]. Anielski and Braaten [39] also examined the impacts of gambling by using an approach they called full cost–benefit accounting, which attempts to overcome the obstacles of CBA. However, like Williams, Rehm and Stevens [32] stated, figures obtained by this approach are not reliable and somewhat arbitrary, and it is not clear how the monetary values for some variables are created. Anielski and Braaten [39] also described many other approaches to study gambling impacts.

In a public health approach, the impacts of gambling, negative and positive, are assessed across the entire severity spectrum of the activity [41]. According to the literature, harms can occur also among those classified as nonproblem gamblers [42]; however, examining only problem or pathological gambling and its impacts on society is still common in economic costing studies [43]. When concentrating solely on problematic gambling, only the tip of the iceberg is observed and gambling harms and its costs to society are underestimated [42]. Additionally, in a public health approach, the positive effects associated with gambling are recognized [17]. In the economic literature, gambling revenues and positive impacts on public services have been observed [32], but fewer studies have examined the positive impacts of gambling on gamblers or their significant others. In a public health approach, the negative impacts of gambling can be assessed by health-related quality of life (HRQL) weights, known as disability weights (DW), which measure the per-person burden of health state on quality of life [44, 45]. DWs have been used to measure intangible social costs of gamblers, but could be also used to discover gambling harms that affect a gambler’s social network. Some studies have attempted to quantify the benefits of gambling by “consumer surplus,” which is the difference between what people would be willing to pay for a product or service versus what they pay [32]. In Australia, the estimated consumer surplus for gambling is AUS$8–$11 billion per year [2]. However, using this arbitrary monetary amount to quantify something that is clearly nonmonetary creates similar problems when trying to place a monetary value on the “social” impacts of gambling [32].

Since the expansion of the gambling market, the question of gambling impacts has piqued researchers and policymakers interest [37]. Despite increased interest in gambling impacts, no consensus has been reached regarding the appropriate theoretical and methodological approach to studying them [32]. A theoretical model is still lacking, although some studies have created basic principles for conducting socioeconomic impact studies. Based on Anielski and Braatan’s socioeconomic impact of gambling (SEIG) framework [39], Williams et al. [32] proposed a simpler categorization of impacts. By doing this, Williams et al. ignored that impacts can be evaluated on different levels, like the individual, family, household, community, regional, and national levels.

Several limitations of earlier gambling impact studies have been highlighted [37, 40], but one major concern has been how to capture and quantify the social impacts [32, 46]. While quantifying the economic impacts is reasonably straightforward (e.g., costs of treating problem gamblers or of preventing problem gambling), this is not the case for social impacts (e.g., invisible costs like the impacts of emotional stress and relationship problems caused by gambling), which cover the major negative impacts from gambling and cannot be evaluated in monetary terms [42].

Thus, studies have mostly ignored social impacts, choosing to measure only the economic costs or benefits that are quite easily quantifiable. This approach, however, presents a very biased view of the situation. There are no established ways to define the social impacts of gambling. Based on Williams et al. [32] social impacts are costs or benefits that are nonmonetary in nature. Walker and Barnett [40] stated that social costs must aggregate societal real wealth, that is, cause harm to someone in the society and benefit no one. They also defined that social cost must be social, rather than personal.

According to these definitions, when a gambler becomes ill because of excessive gambling, their suffering should not be counted as a social cost as long as someone in society gains from this excessive gambling and gamblers do not demand any treatment that would cause costs to society. In our study, rather speaking of social impacts, we use the term nonmonetary impacts (i.e., nonmonetary costs and nonmonetary benefits). Costs and benefits refer to overall negative or positive gambling impacts and not only those with monetary value. We also state that impacts should be examined at the societal, individual, and interpersonal levels.

Compared with existing models, this model combines aspects from costing studies [32, 39] and from gambling harm literature [18, 33–35] making the present model more comprehensive and up to date. The Model emphasizes the public health perspective, which is somewhat different from the one in costing studies. It covers both positive and negative effects of gambling and examines costs and benefits on individual, interpersonal and community/society levels. The model includes a temporal dimension, which refers to the development and severity of gambling behavior. From the public health perspective, it is not presumed that costs and benefits result only from problem gambling; instead we are interested in the whole spectrum of gambling behavior. Costs and benefits can be general, come from problem gambling and/or can have long term effects. In summary, a common and comparable methodology for evaluating the impacts of gambling is necessary [32, 37], and none has been created. Studies have usually concentrated on impacts of problem gambling while ignoring the entire continuum of gambling. Additionally, the emphasis has been on economic costs, whereas most gambling costs are “social.” The benefits of gambling are usually examined at the societal level (e.g., government revenue), and the influence of gambling on gamblers and their significant others are ignored. This debate argues for a conceptual theoretical model based on the gambling impacts literature, where a public health perspective is applied.

## Main text

### Structure of the public health impacts of gambling (PHIGam) model

**Fig. 1:**
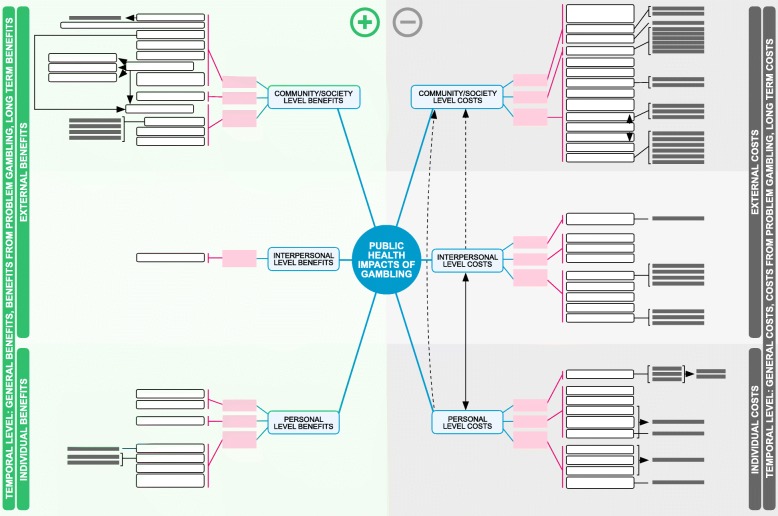
The structure of the Public Health Impacts of Gambling (PHIGam) model

Gambling impacts can be observed at the personal, interpersonal, and community/society levels (Fig. 1). Personal level refers to the gamblers themselves and interpersonal level to people close to the gambler: friends, family and work colleagues. Impacts can be individual or external. Individual impacts induce effects on a personal level to the gambler. External impacts influence the interpersonal and society/community levels and concern those who are not necessarily gamblers themselves. Gambling creates costs and benefits that others must pay for or can exploit. For example, gambler’s increased debt and financial strain affects family members’ lives, and the effects escalating into bankruptcy and homelessness can also be observed on the community level. Thus, it is of utmost importance that impacts are examined on multiple levels. In the model, impacts can be divided into negative and positive. However, impacts can simultaneously be both negative and positive. For example, gambling is linked to increased criminality [44] but can also decrease illegal gambling [45]. Similarly, tourism revenues are positive [46] but on the other hand tourism can increase crime [47].

**Fig. 2:**
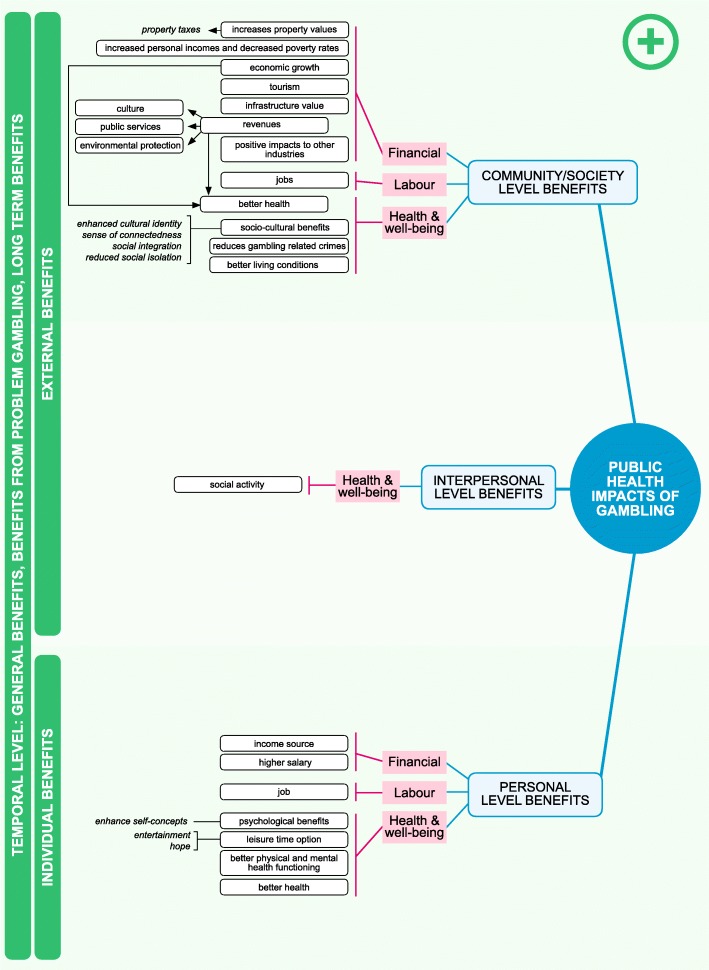
The positive impacts of gambling on personal, interpersonal and community levels

**Fig. 3:**
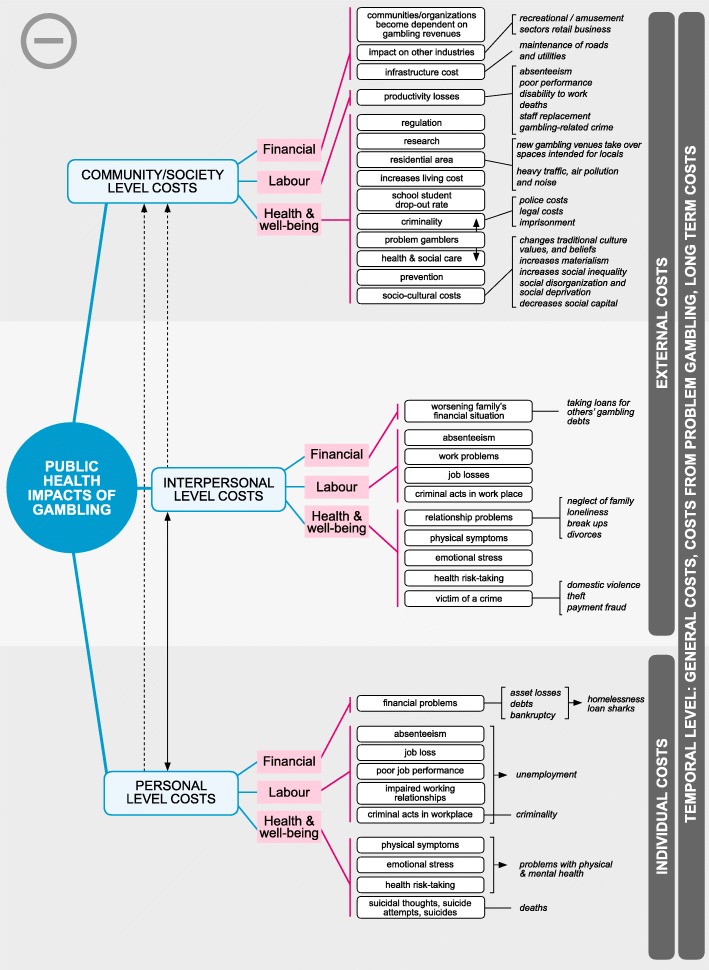
The negative impacts of gambling on personal, interpersonal and community levels

In the model, benefits (Fig. 2) and costs (Fig. 3) are categorized into three classes: financial, labor and health, and well-being. These classes manifest on personal, interpersonal, and societal levels. Financial impacts, for example, include gambling revenues, tourism, impacts on other industries, and infrastructure cost or value change. On the personal and interpersonal levels, financial impacts can be changes in financial situations. Overall, financial impacts contribute to economic activity and economic growth. Labor impacts include gambling effects on work, such as changes in productivity, absenteeism, reduced performance, inability to work, job gains and losses, and unemployment. Health and well-being impacts include the effects that gambling has on physical, psychological, and social health and well-being.

Temporal level refers to the development, severity and scope of the gambling impact. These include general impacts, impacts of problem gambling and long-term impacts of gambling. General impacts usually result from non-problematic (recreational and at-risk gamblers) gambling. For example, gambling can be a leisure time option that takes time and money from other activities. Impacts of problem gambling are severe consequences that materialize on personal, interpersonal and community/society levels. For example, a problem gambler who uses a lot of money on gambling and falls into bankruptcy influences his/her family’s financial situation and the society by creating cost (e.g. social care cost). These long-term effects of problem gambling can materialize even if the person no longer gambles; it can create a change in the life course of an individual, and even pass between generations [18]. On the positive side at society/community level, money spent on gambling increases gambling revenues, which in turn can have positive long-term effects when partly directed to beneficial causes, e.g. public services or environmental protection.

Personal and interpersonal level costs are mostly nonmonetary in nature, including invisible individual and external costs that are general, costs of problem gambling and long-term cost. Some of these invisible costs can turn into visible at the society/community level, for example, when gambler’s family members seek help or treatment. Most of the time, however, these costs remain unrecognized. Society/community level external impacts are mostly monetary and are general costs/benefits, costs/benefits related to problem gambling and long-term cost/benefits.

### Personal level impacts

#### Financial impacts

Financial harms are common, especially among problem gamblers. A survey conducted in Queensland showed that 83% of problem gamblers had experienced gambling-related financial problems [47]. Another study observed that 34% of problem gamblers reported having severe financial difficulties, compared with 23% of at-risk gamblers and 10% of nongamblers [48]. In Finland, almost 8% of the population had experienced some sort of financial harms because of their gambling [49, 50], and among treatment-seeking gamblers, the percentage was 87% [50, 51]. Financial problems can range from escalating harms, such as diminishing savings and borrowing money, to major harms, such as bankruptcy or loss of all valuable possessions [17]. A study conducted among casino visitors observed that 65% of the “problem” players had to turn to others to relieve a desperate financial situation because of their gambling, whereas none of the “social” players had to do so. In addition, 52% of the “problem” players had sold possessions to pay gambling-related debts, compared with 2% of the “social” players [52]. Among treatment-seeking Finnish gamblers, common financial harm included late payment of bills (66%) and turning to income support benefits provided by nongovernmental organizations (32%) [50].

Financial harms have also been observed to be more common in deprived areas [53] and lower socioeconomic groups [19, 54]. Notably, indigenous people are vulnerable to financial problems [55, 56]. Financial risks may also be elevated among problem gamblers with psychotic disorders, because one study showed their increased need for financial assistance [24]. However, causality between financial losses and gambling is not always simple. Factors like ill-health may effect both gambling and poverty, and poverty may lead to problematic gambling and vice versa. However, it is clear, that gambling can intensify poverty [57].

Gambling-related debt develops when borrowed money is spent on gambling [58]. Estimations of average current debt per problem gambler have ranged between USD 2500 to greater than USD 53,000 [2, 32, 59, 60]. For problem gamblers, debts are common, because they were three times as likely to report being in debt compared with nongamblers [48]. Among Finnish treatment-seeking gamblers, 45% had debt problems at one time [50]. Further, the more severe the gambling problem the higher the amount of debt [61].

There is also evidence that some games create more debt than others, because treatment-seeking pathological gamblers playing electronic gaming machines had a higher current and lifetime debt than players of scratch tickets and lotteries [62]. Similar results have been found among treatment-seeking male internet gamblers: patients who bet online had higher debt levels compared with offline gamblers [63]. One study observed that 44% of gamblers regarded as heavy consumers had sometimes taken high-interest instant loans for gambling, and this was more common among female respondents than males [64]. Research has also shown that gambling is a significant contributing factor to indebtedness [65] and often a reason to continue playing [66].

The most serious forms of financial harms because of gambling include bankruptcy and homelessness. These extreme consequences are commonly observed at the point of help-seeking [67]. It is estimated that 10 to 20% of problem gamblers declare bankruptcy [20, 61, 68]. Gamblers who declared bankruptcy were more likely to have more financial, work-related, marital, and legal problems; reported higher rates of depressive and substance use disorders; and were more likely to be daily smokers [68]. Several studies conducted with individuals who are homeless have observed co-occurring problematic gambling [69–74]. Studies have estimated that an average of 12 to 39% of people who are homeless reported having gambling problems [69, 71, 74]. However, similar to other gambling comorbidities, the causal nature of this relationship is difficult to resolve [75]. Gambling may be a risk factor for homelessness: it is often cited as a reason for a lack of housing [67, 75], and 82% of problem gamblers indicated that gambling preceded their homelessness [74].

While the negative financial consequences of gambling are evident, studies have also observed positive financial impacts. For example, in Macao, people working in gambling and related industries earn a higher salary [76], and their nominal wage has increased as a result of casino liberalization [77]. Further, a few studies have shown that for some (especially poker players), although a minority, gambling is a significant income source [78–80].

#### Labor impacts

Although studies have observed that gambling often has a positive effect on employment at the community level [81, 82], only a few studies have examined the positive labor impacts at the personal level and all have considered professional poker players, which represent a minority of people and gamblers.

Problem gambling can affect employment in many ways. Gambling during work causes productivity losses, absenteeism, impaired working relationships [50, 83], and termination of employment [84]. It was observed that almost 40% of problem gamblers reported that gambling had affected their job performance [47], and 61% reported missing work to gamble [83]. Among Finnish treatment-seeking gamblers, 43% evaluated that their work performance got worse due to tiredness or distraction, and among those who gambled within the last 12 months, 1% had used work time to gamble and 0.6% stated that their work performance had decreased [50]. Almost 60% of those experiencing problems with gambling were out of paid work for more than one month and approximately 30% had received some sort of social benefit within the previous year [30]. However, lack of work may not necessarily be because of gambling, although the literature has indicated that problem gamblers were more likely to report poorer work performance. In some cases, problem gambling may lead to criminal acts in the workplace, like embezzlement and stealing goods like office supplies [85].

Employment has critical financial and interpersonal impacts because employment is the primary or major source of household income. Reduced performance in work life can have short- and long-term effects on the life of the individual and their family. Employment also causes effects at the community and societal levels.

#### Health and well-being impacts

Notably, self-reported health decreased with the increasing risk of problem gambling: 57% of nongamblers and 54% of recreational gamblers reported their general health to be good or excellent, whereas 44% of low-risk gamblers, 36% of moderate-risk gamblers, and only 22% of problem gamblers reported good or excellent general health [30]. However, among gamblers aged 65 years and older, it was observed that past-year gamblers reported more often good or excellent well-being compared with nongamblers [86]. Similarly, another study found that gambling contributed independently and significantly to perceived wellness among older Australians [87]. Among older adults, recreational gambling may offer possibilities for increased socialization, community activity, and travel [10, 88], which may have positive effects on health [89].

The health impacts of gambling are related to significant increases in distress [2]. Emotional or psychological distress can be experiences of guilt, anxiety, helplessness, shame, stigma, grief, and self-hatred [50, 90]. It is estimated that 4–6% of those who gambled within the last 12 months had experienced feelings of guilt [30, 50]. Among people experiencing high stress or anxiety, physical changes in an individual’s biochemistry have been noted [91]. Frequent exposure to stress affects an individual’s health outcomes [91], because it has been shown that gambling is associated with heart conditions, high blood pressure, headaches, weight loss, stomach disorders, cardiac arrest, arthritis, indigestion, tachycardia, angina, cirrhosis, and other liver diseases [22, 66, 92]. Notably, problem gamblers were more likely to avoid regular exercise and less likely to seek health care compared with controls [93].

In addition to a lack of regular exercise, problem gamblers had a higher body mass index and were more likely to be classified as obese [93, 94]. They were also more likely to engage in unhealthy lifestyle behaviors, such as watching more than 20 h of television per week, excessive alcohol consumption, and smoking [93]. Other studies have shown strong associations between gambling and substance use: At-risk and problem gamblers had higher rates of tobacco, alcohol, and drug use [30]. Substance use disorders co-occur commonly with problem gambling, because one study showed 28 and 17% of gamblers suffer from alcohol and drug use disorders, respectively [23]. By contrast, 15% of those seeking treatment for alcohol and drug use disorders met the lifetime criteria for problem gambling, and 11% of the current criteria for problem gambling [95]. Smoking is also common among problematic gamblers [96]. Further, problem gamblers were significantly more likely to have smoked more than 100 cigarettes in their lifetime and be current smokers compared with recreational gamblers [30]. Additionally, it has been noticed that among problematic gamblers, 50 to 60% suffered from nicotine dependence [23, 97].

Many studies have shown that problematic gambling is associated with mental health disorders [23, 24, 88]. In New Zealand, 46% of problem gamblers had psychological disorders [30]. Among problematic gamblers, almost 38% had mood disorders and 37% had anxiety disorders [23]. Additionally, substance use has been shown to co-occur with gambling and mental health problems [43, 97–99]. The causality of these health consequences is not clear because gambling can cause negative health outcomes, but is also a coping mechanism to escape physical, emotional, and substance use problems. However, a longitudinal study observed that at-risk and problem gambling predicted future incidents of major depressive disorder, alcohol dependence, and drug use [100].

The gambling literature has also focused on mortality because of suicide [101, 102]. Notably, problematic gamblers have higher rates of suicidal thoughts, suicide attempts, and completed suicides [103–106]. In Finland, 5% of treatment-seeking problem gamblers had attempted suicide, whereas it was 0.1% among the population sample [50]. Studies have also shown a positive correlation among suicidal ideation, suicide attempts, and gambling severity [103, 107, 108]. Heightened risk for gambling-related suicidality is found among youth experiencing gambling problems [109, 110]. A link between gambling and suicide may be explained by excessive debts and escalation of family, legal, and mental and substance-related problems [20, 108]. Notably, the interaction between suicide and gambling is complex, and it would be an oversimplification to assert that gambling causes suicides [17].

The literature also demonstrates the positive effects of gambling. Especially among older adults, recreational gamblers reported better physical and mental health functioning than did older nongamblers [94]. Further, it was proposed that the psychological benefits of gambling may reinforce and enhance seniors’ self-concepts [6]. Additionally, it was stated that among lower socioeconomic groups, gaining pleasure from the hope of a small win and the possibility of making a choice on the use of scarce resources may be important in helping maintain optimism in the face of difficult life circumstances [111]. One of the most obvious positive impacts of gambling is its entertainment value and usefulness as an additional leisure option [112]. Although most adults have engaged in gambling activities, only a minority report that gambling is a very important leisure activity for them or that it has replaced other leisure activities [113].

### Interpersonal level impacts

#### Financial impacts

Gambling affects more people than just the gambler, because an estimate indicates that one person’s gambling problem typically affects 5 to 10 people [2]. Thus, the percentage of people whose lives are negatively impacted by problem gambling may be 3 or 4 times as high than the problem gambling prevalence in the general population [114, 115]. In New Zealand, approximately 30% of adults said they knew at least one person who has/had a problem with gambling, and approximately 8% experienced that someone else’s gambling had affected them personally [30]. Partners and children who share finances with a gambler often experience greater levels of harm [116]. Most commonly reported harms by partners were financial impacts, like increased debt and financial strain [29, 117]. Financial problems can also cause partners to go without daily household items and quality food, cause problems with payments and loss of utilities [118], and further cause the inability to afford medication or treatment [119]. Additionally, partners commonly take loans for someone else’s gambling debts [120]. Children can experience deprivation of essential items and insecurity of material needs [121, 122].

#### Labor impacts

In Australia, 84% of the concerned significant others (CSOs) of people with problem gambling reported that their partners’ gambling had negative impacts on their own employment. Participants with an Asian cultural background had significantly higher employment impacts than their non-Asian counterparts. This was the case also for participants with prior counseling experience [116]. In Sweden, female CSOs reported more sick leave days and months of absence from work because of illness, and male CSOs reported more fear of losing employment and work problems [120].

#### Health and well-being impacts

Financial difficulties can lead to relationship problems, which is common. CSOs experienced a great deal of relationship distress [123], and 96% reported that gambling had negative impacts on their relationships [116]. Among problem gamblers, separation and divorce were more common [25, 115, 124]. In Finland, among the population sample, only 0.1% had experienced separation or ending a relationship because of gambling, whereas among treatment-seeking problem gamblers the it was 10% [50]. Conflict, loss of trust due to dishonesty, concealment of the gambling problem, and need to take responsibility for family and household matters can drive couples to separation or divorce [117].

It is not uncommon that significant others end up as victims of a crime [18]. Petty theft from family members and illicit lending are relative common forms of interpersonal harm. Violence associated with gambling is an extreme form of interpersonal harm. It was observed that pathological gambling increased the odds of perpetrating dating violence, severe marital violence, and severe child abuse even when adjusted for mental disorders [125]. Pathological gambling has also been observed to be associated with homicide in the family [105, 126]. Additionally, among problem gamblers, 63% had been victims or perpetrated intimate partner violence (IPV) [127]. Further, 38% of problem gamblers had experienced physical IPV, and 37% were perpetrators of physical IPV [128]. Additionally, in Asian countries and Asian communities living abroad, high rates of problem gambling and family violence have been observed [129, 130]. Among help-seeking CSOs, 20% were victims of violence, 11% were perpetrators, and 26% were both victims and perpetrators [131]. In Finland 2% of suspected gambling related crimes were intimate partnership violence resulted from gambling problems [132]. This, however, constitute only a small amount of the total partnership violence.

Experiencing isolation and self-blame is common among significant others. Some spouses attempt to conceal partners’ gambling [117]. They felt that the gamblers did not spend sufficient time with them, and they had withdrawn from social life due to their inability to pay for social activities [118]. Thus, CSOs commonly experience isolation and loneliness [120, 133]. Self-blame is another identified pervasive harm, and spouses often feel that they should have been able to prevent their partner from gambling [117].

Gambling is also linked to increased possibilities for social actions [6, 7]. Seniors highlighted the social aspects of their casino visits: they liked having a place to meet and socialize with others [134]. Gambling is also common pastime activity among families [135]; however, this is not necessarily a positive thing because the majority of young people are introduced to gambling by their parents [136].

CSOs experienced poorer physical and mental health than the general population [120, 133, 137]. Symptoms of depression and emotional distress and feelings of melancholy were common [116, 120] as were physical symptoms, like headaches, insomnia, high blood pressure, panic attacks, and feelings of tiredness or exhaustion [138]. CSOs also had problems with their own gambling behavior [139] and with other addictions [120, 137], like risky alcohol consumption for males and daily smoking for females [133].

Children of problem gamblers have an elevated risk of gambling problems [140]. Further, health risk behaviors, such as smoking tobacco, drinking alcohol, and drug abuse are common [141]. These children also have higher a risk for physical and mental health problems and suicide attempts [122, 142, 143]. The effects of parental gambling on children’s overall well-being can be significant, and children can suffer long-term effects because of neglect and uninvolved parenting [121]. Additionally, children whose parents are employed by casinos can suffer from neglect because they are often left at home without much care and can lose contact with their parents [76].

### Community/society level impacts

#### Financial impacts

The introduction of gambling has been associated with increased government revenue and overall economic growth [76, 77, 113, 144]. Governments earn revenue from gambling through several means: the taxation of gambling venues and operations, becoming directly involved in the provision of gambling and receiving its revenue, or by government controlled monopolies, which can deliver various forms of gambling and taxation of gambling winnings [32]. Other studies have observed that gambling does not impact government revenue, and in some cases the impacts have been negative [145, 146]. When new forms of gambling have significant negative impacts on other forms of gambling and states continue to benefit from revenues from the new forms, the net revenues may not change. These revenues can be used for public services, but also to avoid raising taxes and reduce government debt [147].

Some forms of gambling are provided by charitable and community organizations, and these profits are used for their own operation, or the governments’ gambling revenues are earmarked for these groups [32]. However, this scenario can make communities and organizations dependent on gambling revenues [148]. Gambling can also have negative impacts on public services, for example, new forms of gambling in the community can negatively affect charitable gambling revenue through direct competition [149].

Another positive impact of gambling has been increased personal incomes and decreased poverty rates [81, 150, 151]. This was especially observed in Native American communities in the United States [81, 150]. Casino development has also led to an increase in entertainment and recreation facilities, restaurants, shopping places, and bars as well as public performances and exhibitions [151].

The construction of a new gambling venues can increase the physical assets and wealth of a local community [113, 152], especially when infrastructure improvements and construction of complementary businesses (e.g., hotels, restaurants) occur [32, 76, 151]. Notably, increased infrastructural value is not associated with all types of gambling but primarily with those that involve the construction of new venues like casinos [152, 153]. The introduction of machine gaming to Queensland clubs and hotels increased infrastructure value when clubs constructed new building projects and facility improvements [154]. These infrastructure improvements also attract a large number of tourists [151].

Who finances these new gambling venues is important whether these investments can be viewed as cost or benefits; when financed partly or wholly by governments rather than by private developers, investment is construed as more of a “cost,” although the wealth of the local community increases [32]. The costs of public transportation and the required police and fire protection are borne by governments, and the maintenance of roads, electricity, and water supply are usually a government responsibility too [155, 156].

Gambling can also impact other industries. Positive impacts have been observed especially in communities where casinos are located in tourist areas offering other entertainment and sightseeing opportunities and where the casinos’ clients are outside the immediate area and require overnight stays [76, 157–159]. The most common business sectors that benefit from gambling are hotels, restaurants, and other types of entertainment [160, 161]. In addition to the community level, gambling introduction has been shown to increase overall business revenue on a state-wide level [146, 162]. However, some studies have not demonstrated that gambling has significant impacts (either positive or negative) on other industries [113, 163]. Notably, negative impacts as a result of gambling introduction have been reported in the recreational/amusement sectors [164] and for retail businesses [165]. Small ventures are especially likely to have problems with hiring and retaining staff due to the casino expansion, inflation, and increases in shop rents and operating costs [76, 151].

#### Labor impacts

The introduction of a new form of gambling often has positive effects on employment [81, 82]. Gambling that attracts visitors and brings money to a community has potential positive benefits for other business sectors and further employment [76, 166]. This phenomenon is particularly true for the hospitality industry [155]. Employment growth in different types of jobs has helped Macao diversify its economy toward healthier economic development, and foreign investments in casinos have upgraded the city’s international status [76]. Additionally, employment gains were reported for casinos [82]. Automated forms of gambling like electronic gaming machines have only minor impacts on employment [32]. Further, most gambling industry employment is low skilled and low paid; however, a large majority of new gambling employees tend to come from similar low-skilled and low-wage sectors [32, 113]. In Macao, many sectors have reported difficulties recruiting and maintaining staff because people are eager to work for the casino hotels to earn a higher salary [76]. When staff comes from outside the local area, the employment benefits of gambling to a local area can be minimal [32]. In Macao, the government and casino operators chose to import migrant workers to employ a sufficiently skilled workforce [77]. Further, when a situation is examined on a larger than local scope, employment gains of gambling have been minimal or nonexistent [2, 167].

Studies have also found work-related costs because of problem gambling. It is estimated that community cost due to productivity losses varies between US$ 6 million to $39 million [168]. A study conducted in Victoria Australia estimated that productivity loss in the workplace was $323 million [42]. The total cost to the employer of gambling-related staff replacement was $34.6 million, and the unemployment benefit payments $10.8 million. Absenteeism due to gambling problems cost Victoria an estimated $46 million, and the total cost of gambling-related crime was in 2014–15 $22.5 million. The total cost of fatality by suicide due to gambling problems was estimated to be $28.6 million [42].

#### Health and well-being impacts

Governments are typically responsible for regulating gambling operations. Regulations and administration procedures are required to secure functions of the industry and maintain social stability [46]. Thus, increased gambling supply comes with increased regulation costs [168]. In a society where gambling is legal, anyone could suffer from gambling harms. Thus, resources are required to prevent this phenomenon from occurring. A certain amount of public resources also must be allocated to gambling-related professional training and research [46]. One of the major costs of gambling problems borne by governments is the funding for gambling regulations, research, and treatment services, and it is estimated that in 2014–15 the Victorian Government spent at least $52 million on these services [42].

Government revenues are also used to improve public services (e.g., health, education, culture, social security) [152, 153, 169]. In Macao, as a result of casino introduction, more social welfare and benefits have been given to the local people. Additionally, the free education period was prolonged and free medical care and bus transportation for those above 65 years old was offered [76]. Further, public expenditures on environmental protection increased [151]. Additionally, in North American Aboriginal communities, improvements in living conditions and public health have occurred [150, 158]. Enhanced cultural identity has also been reported after casino openings [158]. However, some studies have highlighted how gambling changes traditional Aboriginal culture, values, and beliefs [170, 171], and increasing materialism has also raised some concerns [76, 171]. In Macao, earning money from the casino business was regarded as easier and faster than having a higher education. This phenomenon diminished interest of young people in studying and increased the school drop-out rate [76].

Gambling brings social problems and leads to increased demand for social services [76]. Studies have shown that increased availability of gambling is associated with increased problem gambling rates [155, 172]. A positive relationship has even been observed between casino proximity and problem gambling [173]. Increased gambling opportunities are also associated with increases in social inequality. Higher-income households spend on average much more on gambling, but poorer households lose a higher proportion of their income on gambling [174, 175]. In Germany, the lowest income quintile spent an average of 12% of their net income on gambling, compared with only 2% in the highest quintile. Overall, 50% of gambling turnover was borne by 12.6% of all gamblers [176]. In Finland 50% of gambling turnover come from just 5% of all gamblers [177].

Gambling can have negative effects on quality of life: the introduction of new casinos has increased traffic and cause noise and pollution [76, 149, 151]. Further, casinos can take over areas originally designed for residential and public facilities and conquer green and leisure spaces intended for locals [76]. One study found that quality of life change from gambling is either very modest or negative [178]. Gambling can also increase criminality in several ways [32]. Firstly, by increasing the number of problem gamblers, because problem gamblers are more likely to commit crimes than the general population [110, 179]. Secondly, increasing opportunities for illegal activity and creating venues that sell alcohol and potentially affect alcohol-related offences [32]. The introduction of casinos has been associated with increased violent crime [180] and rates of driving while intoxicated [181]. And thirdly, by increasing the overall number of visitors to the area, because increases in population and tourism contribute to increased crime rates [182]. It is estimated that pathological and problem gambling accounts for $1000 in excess lifetime police costs per person [2]. Study conducted in Sweden proposed that the total court costs for criminal cases caused by gambling would be approximately between $3 and $72 per problem gambler [183]. Cost to the prison system associated with people who are problem gamblers was estimated to be between $51 and $243 million per year [184]. Notably, gambling can decrease the rate of illegal gambling [113].

Property and other living prices have increased faster than average salaries as a result of casino gambling [76]. Further, some studies have shown declines in social capital because of casino introduction [27] and increases in social disorganization and social deprivation [185]. Additionally, the negative consequences of gambling have been linked with social integration, a sense of connectedness, and reduced social isolation [186, 187]. Gambling is also seen as a community activity that brings people together [188].

## Conclusions

The conceptual model developed in this article offers a base on which to start building common methodology for assessing the impact of gambling on the society – a target explicated by, e.g. Walker [37] and Williams and others [32]. In the discussion about the best methodological and theoretical approaches for analyzing the impacts of gambling, the main issue is how to measure the social impacts. Most of the social impacts are nonmonetary by nature and are often difficult to measure and thus ignored in calculations. Similarly, personal and interpersonal impacts have often been excluded from calculation, largely for the same reason as social impacts. Except for the most obvious positive impact of gambling, namely, gambling revenues for communities, studies have often concentrated on the negative side of gambling impacts. The central focus has been on problem gambling; thus, many gambling harms have been ignored although gambling-related harms also occur among those who are not problem gamblers and nongamblers within harms reach, such as significant others and the wider community. These methodological deficiencies are common in the gambling impacts literature and cause a significant bias in current knowledge.

As mentioned earlier, gambling causes external impacts that affect more people than just the gambler. Financial, labor, and health and well-being impacts have been observed at the individual, interpersonal, and community/society levels. For example, gamblers’ increased debt and financial strain affect family members’ lives, and the effects of escalating into bankruptcy and homelessness are also observed in the community. Thus, it is of utmost importance that impacts are examined on separate levels. Additionally, these impacts can have long-term effects and create a change in the life course of an individual, and even pass between generations. Key methodological challenges relate to what portion of impacts are the effects of gambling and how these should be measured. We faced similar methodological challenges when examining the interpersonal and community/society level impacts. Community/society level impacts that are nonmonetary, such as quality of life, social cohesion, and other attributes of social capital, have had less emphasis in studies. These studies have been primarily conducted in North America, and the majority of analyses concerns casino impacts.

Although the PHIGam model attempts to be as universal as possible, it is important to note the context in which gambling takes place is critical when examining gambling impacts. Opening a casino in an area where gambling opportunities have been limited has a greater impact than in area where gambling has been widely available. In the “adaptation hypothesis” [189, 190], it is argued that the negative effects of gambling are higher when gambling or new games are newly introduced in community but tend to diminish over time. However, a more recent study has shown that overall rates of harm stabilized when participation continued to fall and for some groups participation reduced but harms increased [191]. Thus, it is suggested that “adaptation hypothesis” over-simplifies the situation [191, 192]. Financial harms of gambling have been shown to be more common among deprived areas, whereas in Macao, the nominal wages of people working in gambling and related industries has increased because of casino liberalization. Additionally, the type of gambling presented affects impacts because it was shown that some games create more debt than others. Finally, it is important to understand how revenues are derived and disbursed.

The debate leading to the formation of the model on Public Health Impacts of Gambling utilized existing theoretical and empirical literature to form a structure that can be used to locate individual pieces of research. As shown in the Figures, empirical work has largely concentrated on the costs of gambling, especially costs on the community level. The Figures can be used to identify areas where research is scarce: for example, no research was found analyzing financial or labor benefits to the significant others of gamblers. Filling the gaps in knowledge is essential in forming a balanced evidence base on the impacts of gambling. Ideally, this evidence could be the starting point in formulating public policies on gambling.

## Data Availability

Not applicable.

## Abbreviations

CBA: Cost–benefit analysis
CSO: Concerned significant other
DW: Disability weight
HRQL: Health-related quality of life
IPV: Intimate partner violence
PHIGam: Public Health Impacts of Gambling
SEIG: Socioeconomic impact of gambling
